# Autophagy in *Paracoccidioides brasiliensis* under normal mycelia to yeast transition and under selective nutrient deprivation

**DOI:** 10.1371/journal.pone.0202529

**Published:** 2018-08-23

**Authors:** Giselle Ferreira Ribeiro, Caroline Gonçalves de Góes, Diego Santos Onorio, Cláudia Barbosa Ladeira de Campos, Flavia Villaça Morais

**Affiliations:** 1 Laboratório de Biologia Celular e Molecular de Fungos, Instituto de Pesquisa e Desenvolvimento, Universidade do Vale do Paraíba, São José dos Campos, SP, Brazil; 2 Laboratório de Bioquímica, Biologia Celular e Molecular de Fungos, Instituto de Ciência e Tecnologia–Universidade Federal de São Paulo–UNIFESP, São José dos Campos, SP, Brazil; University of Minnesota, UNITED STATES

## Abstract

*Paracoccidioides spp*. is a thermally dimorphic fungus endemic to Latin America and the etiological agent of paracoccidioidomycosis (PCM), a granulomatous disease acquired through fungal propagule inhalation by its mammalian host. The infection is established after successful mycelia to yeast transition in the host pulmonary alveoli. The challenging environment inside the host exposes the fungus to the need of adaptation in order to circumvent nutritional, thermal, oxidative, immunological and other stresses that can directly affect their survival. Considering that autophagy is a response to abrupt environmental changes and is induced by stress conditions, this study hypothesizes that this process might be crucially involved in the adaptation of *Paracoccidioides spp*. to the host and, therefore, it is essential for the proper establishment of the disease. By labelling autophagous vesicles with monodansylcadaverine, autophagy was observed as an early event in cells during the normal mycelium to yeast transition, as well as in yeast cells of *P*. *brasiliensis* under glucose deprivation, and under either rapamycin or 3-methyladenine (3-MA). Findings in this study demonstrated that autophagy is triggered in *P*. *brasiliensis* during the thermal-induced mycelium to yeast transition and by glucose-limited conditions in yeasts, both of which modulated by rapamycin or 3-MA. Certainly, further genetic and *in vivo* analyses are needed in order to finally address the contribution of autophagy for adaptation. Yet, our data propose that autophagy possibly plays an important role in *Paracoccidioides brasiliensis* virulence and pathogenicity.

## Introduction

Paracoccidioidomycosis (PCM) is a systemic granulomatous disease, geographically confined to Latin America, caused by two species of the genus *Paracoccidioides*, *P*. *brasiliensis* and *P*. *lutzii* [1,2]. Subsequently the inhalation of fungus spores by a suitable host, the mycelia (infective form) undergo a thermal-induced differentiation into the yeast parasitic form in the host’s lungs [3]. Once inside the host, the fungus experiences several stresses, such as high temperature, host immune system response and nutrient deprivation [4,5], particularly glucose and amino acids, as suggested by Parente-Rocha et al [6] and Tavares et al [7] in their proteomic and transcriptomic analyses of *P*. *brasiliensis* during macrophage interaction *in vitro*.

Macroautophagy, the prevailing form of autophagy, is one well-known mechanism involved in the response to nutritional deprivation [8]. It aims at counterbalancing the nutrient-scarce environment by reducing the energetic demand of biochemical processes in the cell, as well as providing nutrients to the cell after digesting its own contents inside autophagosomal vesicles [9,10]. Under normal conditions, catabolism and anabolism are two antagonistic mechanisms involved in the maintenance of cells in eukaryotes, both of which controlled by the TOR signalling pathway [11,12]. Macroautophagy (herein named autophagy) represents the cell catabolism of the biological macromolecule triggered when TOR is not active, whereas the activation of the TOR signalling pathway stimulates processes, such as protein synthesis and cell survival, and drives the increase of cell size and mass required to cell growth in an environment that provides enough nutrients to support cells to complete mitosis [11,13].

TOR, a serine/threonine-directed protein kinase found in all eukaryotes, is currently called mTOR, or mechanistic TOR. It constitutes the catalytic subunit of two distinct protein complexes known as mTOR Complex 1 (mTORC1) and mTOR Complex 2 (mTORC2), both of which hold some overlapping functions [14]. They function preponderantly as a sensor indicating the suitability of the environment’s nutritional conditions necessary for the accomplishment of cellular division in response to endocrine stimuli [15]. Specifically, mTORCs control the balance between anabolism and catabolism by stimulating the biosynthesis of macromolecules necessary for cell growth and proliferation, maintaining inactive catabolic pathways such as those leading to autophagy and protein expression of the ubiquitin-proteasome system, thus preventing the activation of pathways which control different types of programmed cell death [16].

Despite the most well-known TOR inhibitor, rapamycin, which also originated the name of this protein kinase (**T**arget-**O**f-**R**apamycin), directly inhibiting mTORC1, it does not suppress mTORC2. While the chronic use of rapamycin may also lead to the inhibition of mTORC2, this effect seems to be more related to the impossibility of assembling new mTORC2 complexes than to the direct inhibition of the formed ones [17–19]. The difference in sensitivity to rapamycin allows differentiating the action of each complex in more defined modules of cellular responses to growth factors. Whereas mTORC2 is primarily involved in the control of cell survival and proliferation, mTORC1 regulates cell metabolism and growth while suppressing autophagy [16]. Thus, it can be said that the effect of rapamycin on eukaryotic cells is mostly associated with mTORC1 and this effect is often related to the stimulation of catabolism and the appearance of autophagic vacuoles by autophagy derepression.

Nevertheless, autophagy is not activated exclusively by nutrient deprivation or in the absence of survival factors. Eukaryotic cells can induce autophagy in several other adverse conditions, such as temperature variation, hypoxia, accumulation of damaged organelles, aggregation of proteins and oxidative stress [20,21]. Essentially, autophagy is a mechanism in which cells adapt to a noxious environment in order to fit a non-efficient cell metabolism to the surrounding demand, particularly when it had changed abruptly. In any case, autophagy allows a rapid change of cell components while it generates nutrients by recycling organelles and other cellular contents to regulate cell adaptation, and then survival, growth, differentiation, and even death [22]. In microorganisms, autophagy clearly has a role in adapting to the environment. While inhibiting autophagy might be deleterious to cells when associated with environmental stresses, stimulating it may accelerate rapidly-dependent cellular processes (turnover) of macromolecules and organelles.

In filamentous fungi, many studies have demonstrated the importance of autophagy for differentiation, adaptation, development and reproduction [23–28]. Studies have shown that autophagy is implicated in the regulation of mitochondrial functions via aerobic respiration of *Aspergillus nidulans* under carbon deprivation [29], in growth halting, hyphal development and vacuolization of *Aspergillus oryzae* [30], and in the growth of aerial hyphae, conidia and adequate asexual differentiation of *Magnaporthe oryzae* [31]. In *P*. *brasiliensis*, apoptosis and autophagy-like mechanisms have recently been implicated in a cyclopalladated 7a-mediated cell death [32]. Gontijo et al [33] showed that 21 of 34 autophagy-related genes described in *Saccharomyces cerevisiae* are present in a basidiomycete, *Cryptococcus neoformans*, and in *Candida albicans* and *Aspergillus fumigatus*, both ascomycetes. Therefore, there is an increasing evidence that autophagy is indeed ubiquitous in the Fungi Kingdom. Considering all the examples found, autophagy allows the cells to adapt to environmental constraints. The recycling of cellular components by autophagy may also occur as part of a fine-tuning to reach the cell physiological homeostasis that is far from being essentially severe or often fully evident.

Several compounds can inhibit autophagy. For instance, 3-methyladenine (3-MA), wortmannin, and LY294002 have been described as compounds capable of suppressing autophagy in its early stages through the inhibition of class III phosphatidylinositol 3-kinases (PI3K) [34–37]. Moreover, chloroquine (CQ) and bafilomycin A1 have also been described as autophagy inhibitor compounds, both of which act on the suppression of lysosomal function, thus, blocking later stages of autophagy [38,39]. Recently, a new compound, MHY1485, has been depicted as an inhibitor of autophagy in mammalian cells by promoting TOR activation and preventing fusion of autophagosomes and lysosomes, though its use has not been characterized in plants and fungi [40].

Studies about the mechanisms responsible for triggering autophagy in *Paracoccidioides spp*, as well as the occurrence of the autophagic process itself, have only been initiated very recently with a single demonstration made by Arruda et al [32] that mechanisms resembling autophagy-like cell death may be behind the cyclopalladated 7a compound killing of *P*. *brasiliensis*. This work aims at demonstrating that autophagy is an active process triggered in *P*. *brasiliensis* in response to abrupt environmental changes, such as a restrictive temperature or a sudden decrease of glucose availability. Specifically, we present evidence that autophagy may be involved in adaptation processes required by *P*. *brasiliensis to* overcome the thermal-induced dimorphism as well as to circumvent nutrient restrictions. Advances in the knowledge of mechanisms controlling autophagy in *P*. *brasiliensis* may reveal new promising candidate molecules important for pathogenicity and virulence in this and other fungi.

## Materials and methods

### Microorganism and growth condition

*P*. *brasiliensis* yeasts, isolate 18 (Pb18), were grown in synthetic dextrose medium, SD (0.17% yeast nitrogen base w/o amino acids and ammonium sulphate (Difco), 2% glucose (Difco), 0.5% casamino acids (Difco), 0.5% ammonium sulphate (Synth), pH 4.5). The temperature was kept at 25°C or 36°C, under constant agitation, to grow mycelia or yeast, respectively.

After 5–7 days of growing at a constant temperature of 36°C, yeasts were collected and washed with PBS. The viable cell concentration was adjusted to 1x10^7^ yeasts ml^-1^, by using a Neubauer chamber and methylene blue was used as a dye. For Pb18 yeasts experiments, 1x10^6^ yeasts.ml^-1^ were inoculated in different culture media: SD (synthetic medium) as control, SD-G (SD without glucose), SD 0.2% (SD containing 0.2% glucose), SD+R (SD containing 0.2 μg.ml^-1^ rapamycin), SD+3 (SD containing 10 mM 3-methyladenine), and incubated at 36°C, under constant agitation for 6 days.

For mycelia to yeast transition experiments, mycelia, grown for 10 to 15 days at 25°C, were inoculated in SD medium and SD+R, and later incubated at 36°C for up to 24 hours.

### Quantification of the enlarged structures in the hyphal tips of *P*. *brasiliensis* during mycelia to yeast transition

Mycelia to yeast transition cells of Pb18 treated in the media SD+R and SD were collected, fixed in paraformaldehyde (4% in PBS, pH 7.2), and photo-documented using a Leica DMLB microscope, coupled to a Leica DFC310 FX camera. The values were described as percentages of enlarged tips over total tips counts. For each experimental condition, at least 200 hyphal tips were counted and distributed between 7 to 10 images taken from different visual fields. According to Campos et al [41], each visible hyphal tip was observed and classified in: hyphae showing enlarged tips with buds (HEB); hyphae with enlarged tips (HE); and hyphae without enlarged tips (H).

### Staining of autophagic vesicles with monodansylcadaverine

Monodansylcadaverine (MDC) was used for staining autophagic vesicles [36]. Mycelia or yeasts of Pb18 growing under different conditions were harvested by centrifugation, washed three times with PBS (pH 7.2), and incubated for 15 minutes with 50 μM MDC in the dark at 37°C. After three washes with PBS, the cells were then visualized and photo-documented under fluorescence by using a Leica DMLB microscope, coupled to a Leica DFC310 FX camera. MDC labelling produces green fluorescence (excitation/emission at 488/505 nm) of autophagic vesicles in the cells.

### In silico analysis

34 autophagy-related genes (ATG) and the 33 non-ATG genes related to autophagy were obtained from the *Saccharomyces* Genome Database (SGD) [42] in order to identify which genes related to autophagy described in *Saccharomyces cerevisiae* would be present in the genomes of *P*. *brasiliensis* and *P*. *lutzii*.

The obtained sequences were used as a query in ‘*tblastn’* search on the online BLAST web interface provided by NCBI [43,44]. All the searches were done using *Paracoccidioides* (taxid:38946), *Aspergillus* (taxid:5052), *Cryptococcus* (taxid:5206) and *Candida* (taxid:1535326) database. All *Paracoccidioides spp*. gene sequences were validated by performing a search for each one as a query in the SGD.

### Dry weight assay

Pb18 yeasts grown at 36°C in SD-G, SD0.2%, SD, SD+R were recovered after 3 and 6 days had their growth estimated considering their dry weight. Triplicates of 1.5 ml of each culture were collected, and the cells were subsequently washed in 100% ethanol, centrifuged and dried at 60°C. The dry pellet weight was calculated by the subtraction of the weight of the centrifuge vials with the dry cells by the empty ones. The results were plotted in a graph.

### Cell viability assay

For six consecutive days, 1.0 ml of each Pb18 yeast culture (SD-G, SD0.2%, SD, SD+R and SD+3) was submitted to serial dilutions (1/2, 1/4 and 1/8). Following, 10 μl of each dilution, as well as the starting culture, were inoculated into Petri dishes containing solid YPD medium (1% Dextrose, 0.5% Yeast Extract, 0.5% peptone and 1.8% bacteriological agar). After 3 and 6 days of incubation at 36°C, the yeast growth was photo documented using a digital camera.

### Statistical analysis

Statistical analyses were performed using ANOVA test. The results *p*<0.05 or *p*<0.01 were considered statistically significant. Data are either the means or representative results of at least three similar repetitions, each one performed in triplicate.

## Results

In the two species of *Paracoccidiodes*, we used BLAST to identify 21 of the 34 autophagy-related genes that were initially described in *S*. *cerevisiae*. This matches the number identified by Gontijo et al [33] for *Candida albicans* and *Aspergillus fumigatus* (Table 1).

**Table 1:** Genes involved in autophagy described in the Saccharomyces Genome Database and their respective homolog one in *Paracoccidioides* species.

*Genes*	SGD SN*	*NCBI Reference Sequence*	
	*S*. *cerevisiae*	*P*. *brasiliensis*	*P*. *lutzii*
***ATG 1***	YGL180W	XM_010759982.1	XM_015844907.1
***ATG 2***	YNL242W	XM_010762092.1	XM_015845428.1
***ATG 3***	YNR007C	XM_010759428.1	XM_002797908.2
***ATG 4***	YNL223W	XM_010763168.1	XM_002789114.2
***ATG 5***	YPL149W	XM_010759117.1	XM_002791664.2
***ATG 6*** ***(VPS 30)***	YPL120W	XM_010760204.1	XM_002793231.2
***ATG 7***	YHR171W	XM_010765002.1	XM_015843967.1
***ATG 8***	YBL078C	XM_010765174.1	XM_002790080.2
***ATG 9***	YDL149W	XM_010762134.1	XM_002792896.1
***ATG 10***	YLL042C	—	—
***ATG 11***	YPR049C	XM_010758143.1	XM_002796055.1
***ATG 12***	YBR217W	XM_010761218.1	XM_002797321.2
***ATG 13***	YPR185W	XM_010760793.1	XM_002797350.2
***ATG 14***	YBR128C	—	—
***ATG 15***	YCR068W	XM_010759052.1	XM_002791514.2
***ATG 16***	YMR159C	XM_010762766.1	XM_002795707.2
***ATG 17***	YLR423C	XM_010764246.1	XM_002797570.1
***ATG 18***	YFR021W	XM_010762746.1	XM_015844633.1
***ATG 19***	YOL082W	—	—
***ATG 20***	YDL113C	XM_010758660.1	XM_002797438.2 XM_010764341.1
***ATG 21***	YPL100W	XM_010762746.1	XM_015844633.1
***ATG 22***	YCL038C	XM_010765004.1	XM_002797697.2
***ATG 23***	YLR431C	—	—
***ATG 24 (SNX4)***	YJL036W	XM_010758660.1	XM_002791067.2
***ATG 27***	YJL178C	—	—
***ATG 29***	YPL166W	—	—
***ATG 31***	YDR022C	—	—
***ATG 32***	YIL146C	—	—
***ATG 33***	YLR356W	—	—
***ATG 34***	YOL083W	—	—
***ATG 36***	YJL185C	—	—
***ATG 38***	YLR211C	—	—
***ATG 39***	YLR312C	—	—
***ATG 40***	YOR152C	—	—
***ATG 41***	YPL250C	—	—
***AMS1***	YGL156W	XM_010761441.1	XM_002796780.2
***APE1/APE4***	YKL103C YHR113W	XM_010764556.1 XM_010761450.1	XM_002793887.2 XM_002796759.2
***BET3/ BET5***	YKR068C YML077W	XM_010761106.1	XM_015847804.1
***CUE5***	YOR042W	XM_010759010.1	XM_015843993.1
***GTR2***	YGR163W	XM_010763723.1	XM_015846543.1
***GYP6***	YJL044C	XM_010759156.1	XM_015846565.1
***IRS4/TAX4***	YKR019C YJL083W	XM_010762511.1	XM_002795701.2
***MON1***	YGL124C	XM_010761281.1	XM_015844238.1
***PEP4***	YPL154C	XM_010758131.1 AY278218.1	XM_002796032.2
***PEP5***	YMR231W	XM_010761933.1	XM_015845493.1
***PTC6***	YCR079W	XM_010763747.1	XM_015845540.1
***SEC16***	YPL085W	XM_010762055.1	XM_002793635.1
***SEC17***	YBL050W	XM_010764217.1	XM_002791922.1
***SEC2***	YNL272C	XM_010763227.1	XM_002792074.1
***SEC4***	YFL005W	XM_010757981.1	XM_002796446.2
***SNX3***	YOR357C	XM_010760051.1	XM_002794181.1
***TRS130***	YMR218C	XM_010764348.1	XM_002789350.1
***TRS20***	YBR254C	XM_010762659.1	XM_002788977.2
***TRS23***	YDR246W	XM_010757381.1	XM_015844418.1
***TRS31***	YDR472W	XM_010763964.1	XM_015844671.1
***TRS33***	YOR115C	XM_010765121.1	XM_002789624.2
***UTH1***	YKR042W	XM_010764236.1	XM_002797590.2
***VPS15***	YBR097W	XM_010760756.1	XM_002794317.2
***VPS34***	YLR240W	XM_010764380.1	XM_015845153.1
***YPT1***	YFL038C	XM_010765286.1	XM_015846400.1
***YPT31/YPT32***	YER031C/ YGL210W	XM_010765342.1	XM_015846423.1

* SGD Systematic Name

Considering the 34 *ATG*-genes described in *S*. *cerevisiae*, there were 9 missing genes among the species of *Paracoccidioides*, *C*. *albicans* and *A*. *fumigatus* (*ATG10*, *ATG14*, *ATG23*, *ATG31*, *ATG32*, *ATG34*, *ATG36*, *ATG39*, *ATG40* and *ATG41*); 2 shared genes between the species of *Paracoccidioides* and *C*. *albicans* (*ATG21*, *ATG24*); 3 shared genes between the species of *Paracoccidioides* and *A*. *fumigatus* (*ATG11*, *ATG17* and *ATG22*); 1 shared gene between the species of *C*. *albicans* and *A*. *fumigatus* (*ATG27*); 1 gene present only in *C*. *albicans* (*ATG33*); 3 genes present only in *A*. *fumigatus* (*ATG19*, *ATG29* and *ATG38*). The other remaining genes were present in the genome of these three microorganisms (*ATG1*, *ATG3*, *ATG3*, *ATG4*, *ATG5*, *ATG6*, *ATG7*, *ATG8*, *ATG9*, *ATG12*, *ATG13*, *ATG15*, *ATG16*, and *ATG18*).

In addition to the so-called *ATG* genes, there are other 33 genes (non-*ATG* genes) known to be involved in the autophagy process in *S*. *cerevisiae*; at least 20 of which were present in the species of *Paracoccidioides* (*BET3* / *BET5*, *CUE5*, *GTR2*, *GYP6*, *IRS4* / *TAX4*, *MON1*, *PTC6*, *SEC16*, *SEC17*, *SEC2*, *SEC4*, *SNX3*, *TRS130*, *TRS20*, *TRS23*, *TRS31*, *TRS33*, *UTH1*, *YPT1*, *YPT31* / *YPT32*).

To investigate whether the autophagic process would act on the adaptation of the fungus in regard to the restrictive temperature of 36°C during the dimorphic transition process, we initially sought to observe the emergence of monodansylcadaverine-labelled (MDC-labelled) vesicles typically found in autophagy during the early stages of mycelium to yeast (M-Y) transition.

**Fig 1:**
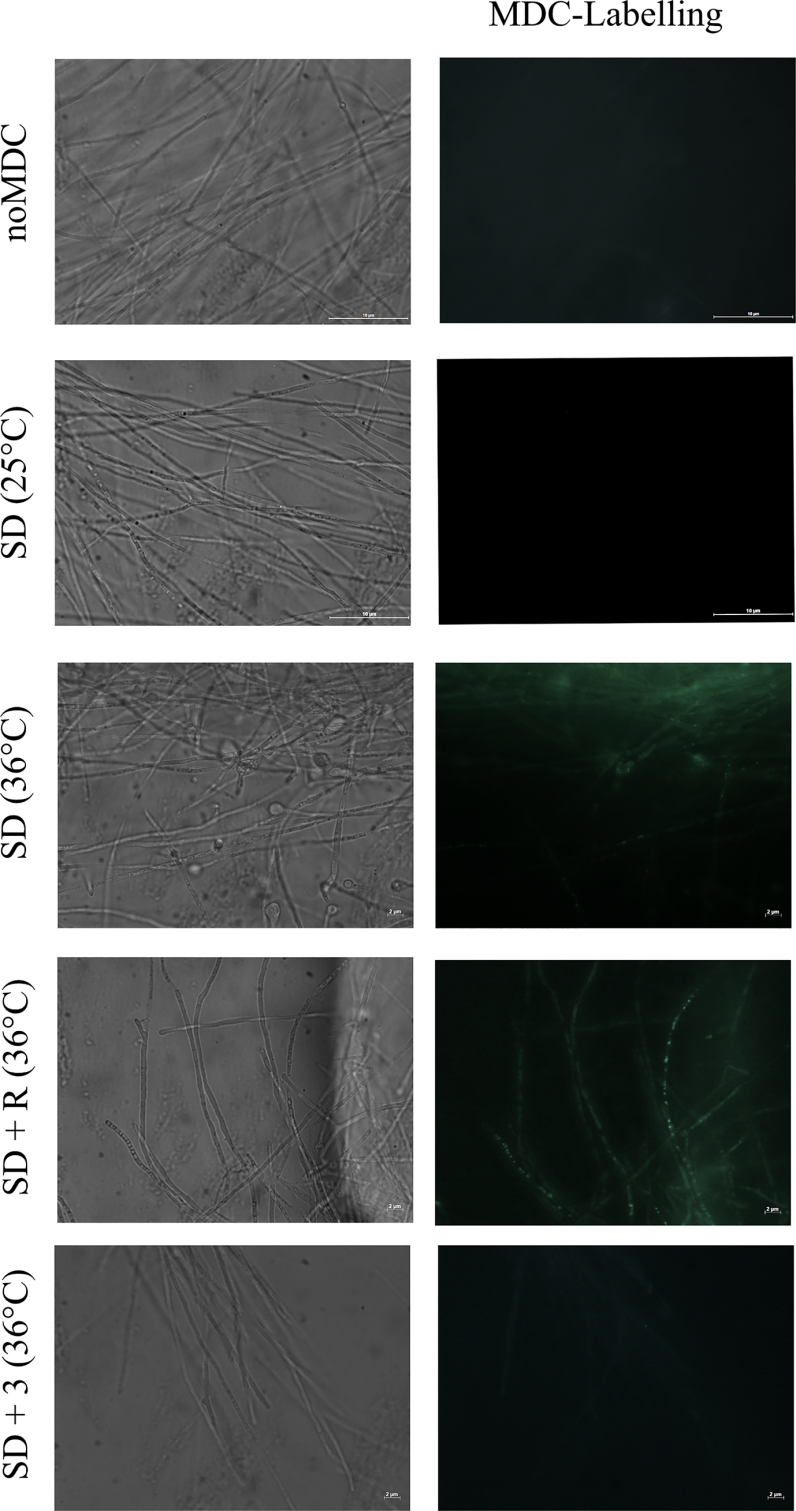
MDC-labelled vesicles after 24 hours of M-Y transition can be modulated by rapamycin or 3-MA. Pb18 mycelia were incubated in Synthetic Dextrose medium as control (SD) at 25°C or 36°C or in SD medium containing 0.2 μg.ml-1 rapamycin (SD+R) or 10 mM 3-MA (SD+3) at 36°C. After 24 hours, fungi were incubated with MDC at 50 μM for 15 minutes, washed three times in PBS pH 7.2 and then immediately analyzed under the fluorescence microscope as aforementioned in Materials and Methods. Autophagic vesicles are stained in green. As control was used Pb18 mycelia after 24 hours of mycelium to yeast transition without MDC labelling. Scale bar at 2 μm or 10 μm.

It was observed that Pb18 mycelia grown under standard conditions (Synthetic Dextrose medium—SD) rarely showed MDC-labelled autophagic vesicles (Fig 1, SD-25°C). Nonetheless, an increase in the number of MDC-labelled hyphal structures was observed at 24 hours subsequent to the onset of M-Y differentiation induced by increasing the temperature from 25°C to 36°C (Fig 1, SD-25°C vs. SD-36°C). Although the increase in temperature clearly induced the emergence of autophagic-like vesicles during the regular thermal-induced dimorphism, it was still a mild effect since few cells showed MDC-labelled vesicles.

**Fig 2:**
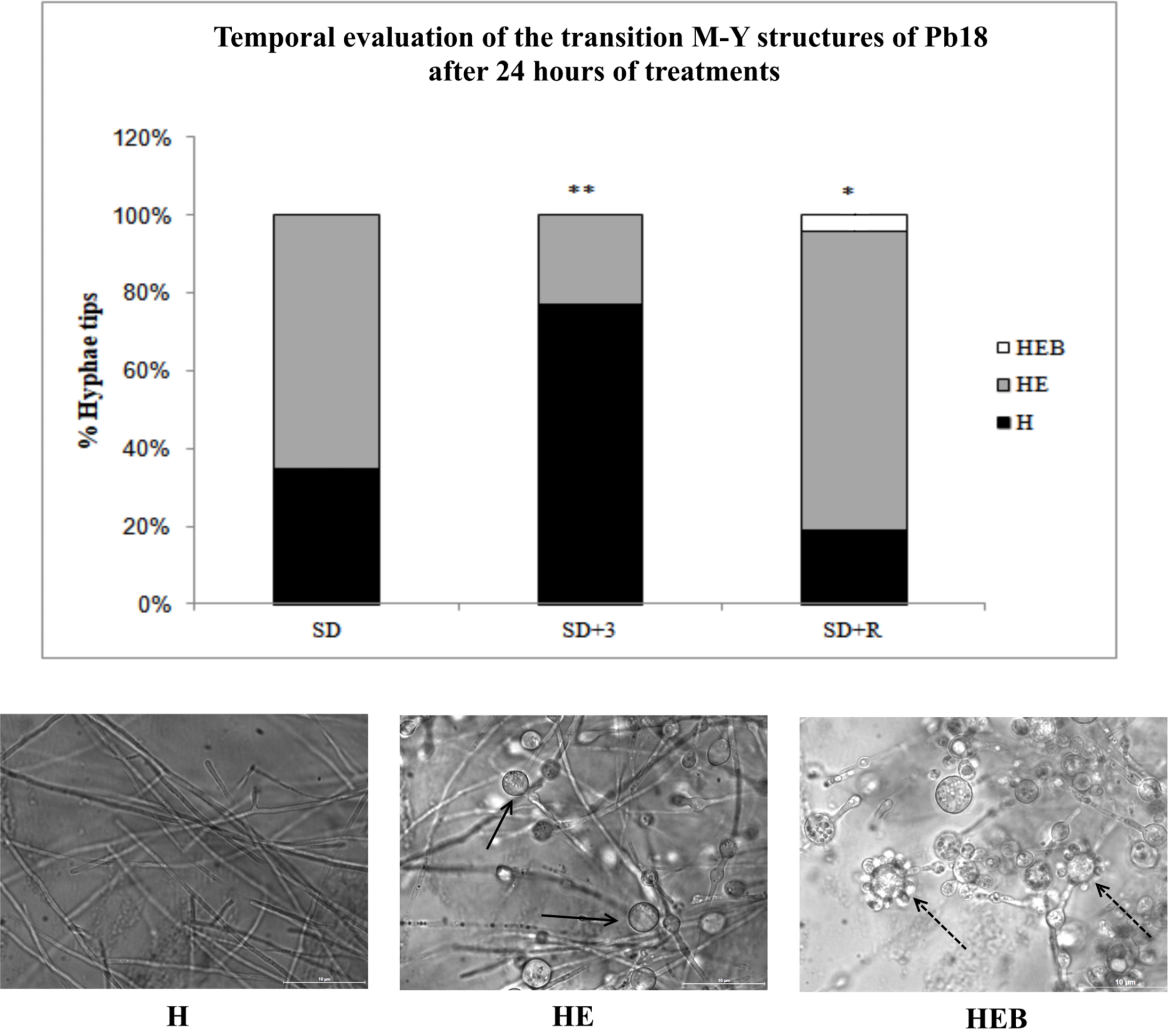
M-Y transition showed different rates for the typical transition structures along the first 24 hours. (A) Graphical summary of the rate of morphological changes in hyphal tips during the first 24 hours of progression through the M-Y transition at 36°C in the absence of modulators (SD) or presence of 0.2 μg ml^-1^ rapamycin (SD+R) or 10 mM 3-MA (SD+3). (B) H—Hyphae without enlarged tips; HE—Hyphae with enlarged tips; HEB—Hyphae showing enlarged tips with bud. The arrow indicates HE structure, and the dashed arrow indicates HEB structure. Scale bar = 10 μm. * means *p*>0.05 and ** *p*>0.01.

Despite its apparent low frequency, autophagy was required for dimorphism when the 3-methyladenine (3-MA) autophagy inhibitor abolished the emergence of MDC-labelled vesicles (Fig 1, SD + 3) and decreased the incidence of hyphae with enlarged tips (HE) that are typically found during the very early phase of the M-Y transition (Fig 2A). In contrast, rapamycin, a TOR inhibitor and an autophagy-inducing drug, enhanced the emergence of MDC-labelled vesicles during M-Y transition (Fig 1, SD + R), well above the mild induction of autophagic-like vesicles observed (Fig 1, SD 36° C). Interestingly, rapamycin also hastened the emergence of HEB hyphae showing enlarged tips with buds (HEB) after the onset of dimorphism. That is, the typical transition structures, such as the HE (Fig 2B, arrow) and HEB (Fig 2B, dashed arrow), were significantly higher in rapamycin-treated than in non-treated mycelia (SD+R and SD, respectively).

**Fig 3:**
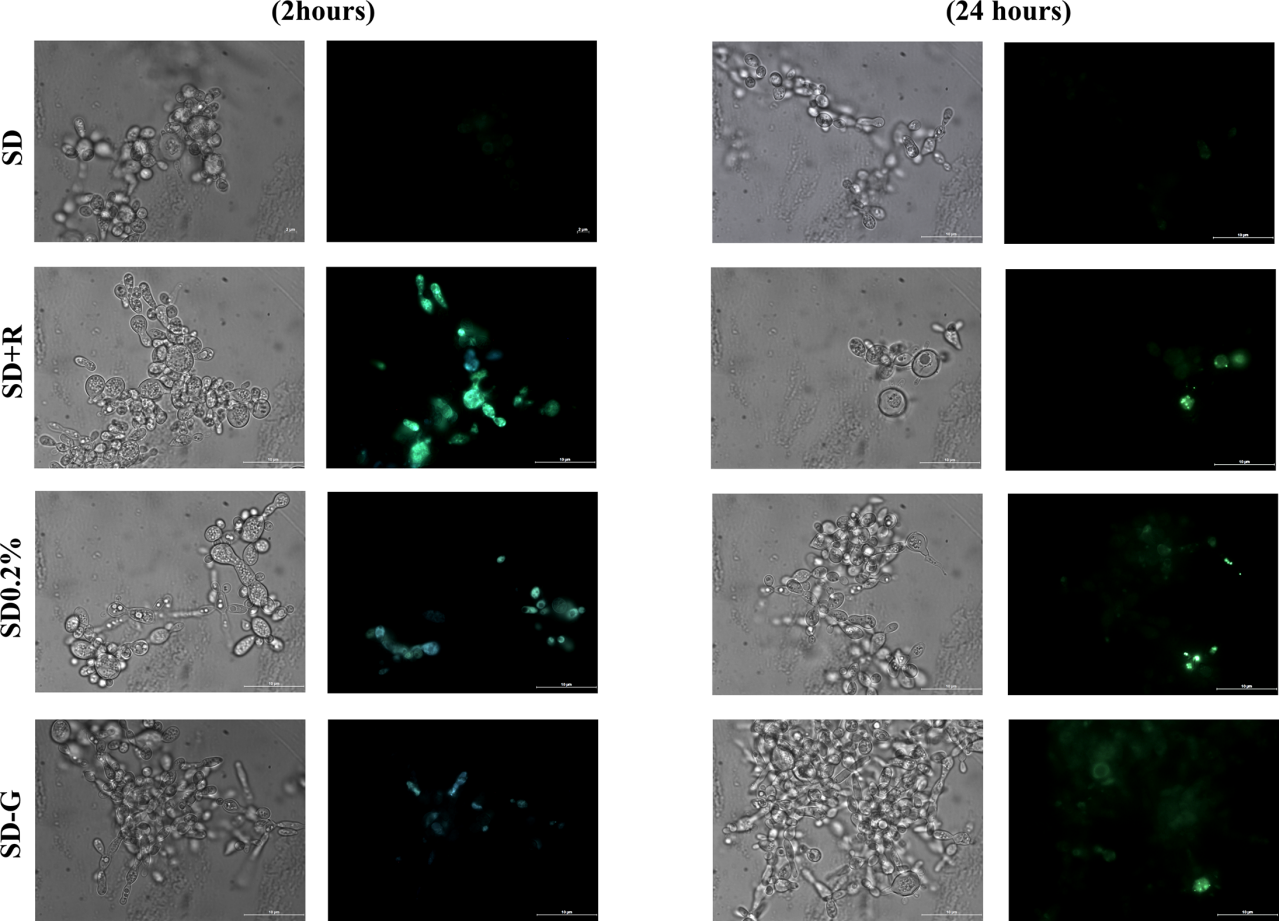
MDC-labelled vesicles are induced by rapamycin and glucose deprivation in yeast cells. Pb18 yeasts growing in SD medium for 4–5 days were washed and incubated (1x10^6^ cell ml^-1^) in Synthetic Dextrose medium as control (SD), or in SD medium containing 0.2 μg ml^-1^ rapamycin (SD+R); SD medium containing 0.2% glucose (SD0.2%); SD medium without glucose (SD-G). After 2 and 24 hours at 36°C, cells were incubated with MDC at 50 μM for 15 minutes, washed three times in PBS pH 7.2 and then immediately analyzed by fluorescence microscopy as described in Materials and Methods. Autophagic vesicles are stained in green. Arrow indicates yeasts entirely full of buds. Scale bar at 10 μm.

We also aimed at showing that autophagy is a universal adaptation process that *P*. *brasiliensis* makes use of under various circumstances, either as a mycelium or as a yeast. Firstly, yeasts can also readily switch on the mechanisms of autophagy induction when in the presence of rapamycin. After 2 hours of TOR inhibition with 0.2 μg ml-1 rapamycin, an expressive increase in the number of MDC-labelled vesicles was observed in the cytoplasm of cells (Fig 3, SD + R). This data unveils that the components (or machinery) necessary to trigger autophagy are likely present in yeast and that they are also kept inactive by the rapamycin-sensitive TOR in a conserved manner in *P*. *brasiliensis*, as it is found in other eukaryotes.

It is important to highlight that rapamycin-treated yeasts (SD+R) displayed a higher number of buds emerging from the mother cells (Fig 3, arrow), as an indication that rapamycin might favour proliferation and budding.

**Fig 4:**
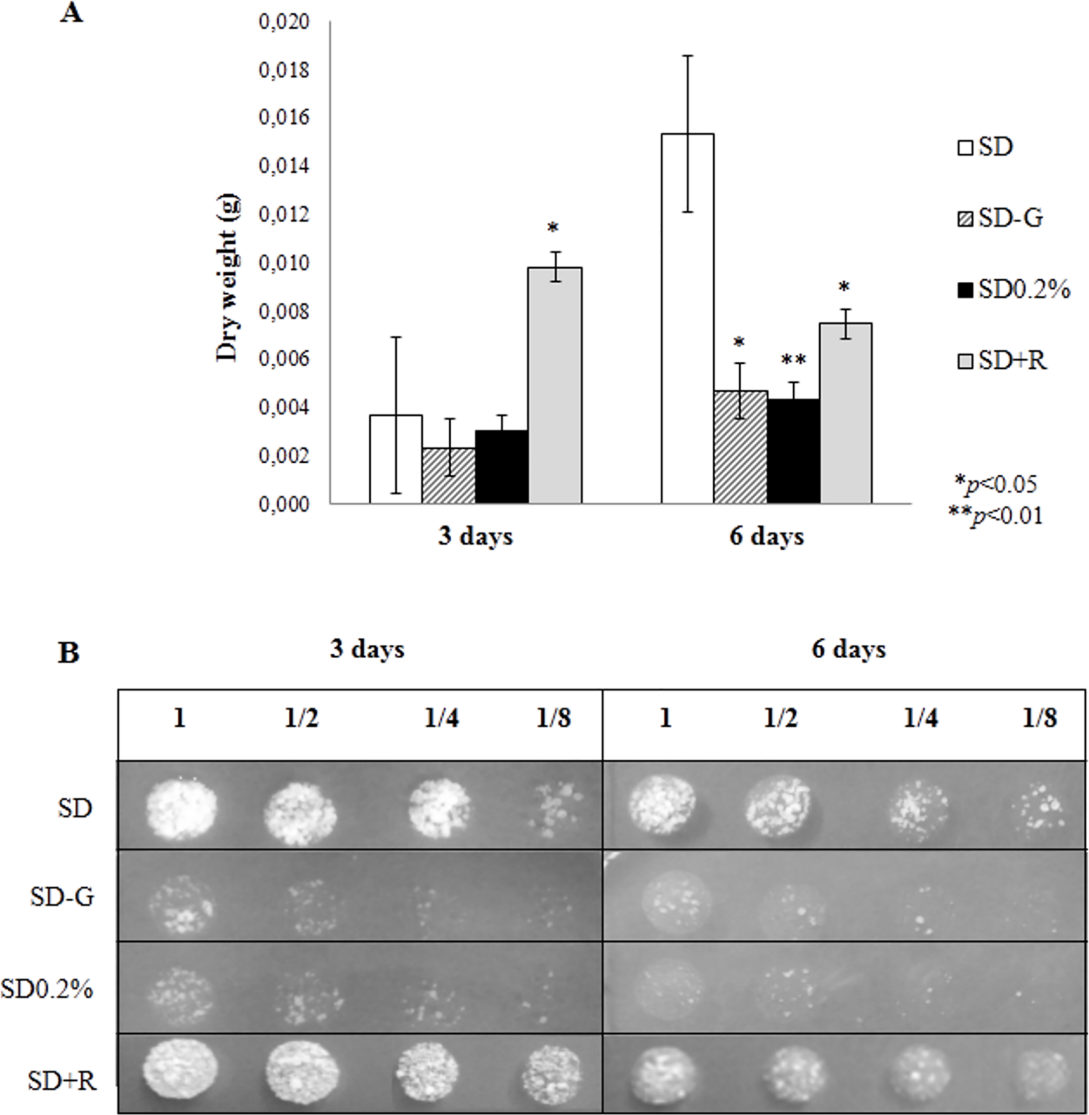
Total dry weight and yeast cellular viability of Pb18. (A) Chart showing total yeast dry weight of yeasts of Pb18 in the different culture treatment. (B) Image of plated Pb18 yeasts after serial dilution (1, 1/2, 1/4 and 1/8), incubated for 3 days, in solid YPD medium. Results were statistically significant in comparison to the control (SD), with *p<0*.*05* * or *p<0*.*01* **.

In fact, the appearance of typical MDC-labelled autophagic vesicles observed following the treatment with rapamycin correlates with an increased yeast proliferation up to 3 days being cultured (Fig 4A), but not afterwards. At 6 days, the chronic treatment of yeasts with rapamycin inhibits proliferation, probably leading to the already described paradoxical effect, resulting from the late inhibition of mTORC2 and consequent loss of cellular viability [18,19], as seen in Fig 4B.

Cells grown under either low glucose medium (SD 0.2%) or glucose-deprived medium (SD-G) showed an increase in the number of MDC-labelled autophagic structures in their cytoplasm (Fig 3). Although it was only a discrete increase in the incidence of MDC-labelled vesicles, the autophagy triggered in yeasts under glucose limitation can be associated with the adaptation in which yeasts undergo inside macrophage's phagosome, a nutrient-limited environment.

Still, even if autophagy played a crucial role in these glucose starving cells (SD0.2% and SD-G), it is possible that autophagy might not be sufficient to sustain cellular proliferation or viability (Fig 4A and 4B), and eventually some other stimulus or cell response may be required to hold yeast cells inside the host.

## Discussion

The mycelium to yeast transition in *Paracoccidioides spp*. is known to be directed by temperature. When the fungus in its mycelial form is subjected to 36°C, it turns into yeast form causing pulmonary and/or disseminated disease [45,46]. Nonetheless, it is important to highlight that not only is temperature variation faced by the fungus during the process of finding a host organism; it also deals with differences in nutritional availability [6,47].

After reaching the pulmonary alveoli, conidia and parts of the fungus mycelium start transitioning into yeast [45]. In addition, it is possible to find yeasts with shoots inside the autophagic vacuoles in the first 18 hours after the initial infection [45]. During this process, the fungus undergoes nutritional stress since it is believed that the mucosal surface of the lungs and the interior of macrophages are poor in nutrients, such as glucose and amino acids [5,48]. Hence, mechanisms involved in cell remodelling, adaptation, and differentiation must be induced in the fungus in order to allow its thermal dimorphic transition [47].

In this work, mycelium of Pb18 grown in nutrient-rich medium and submitted to the dimorphic transition displayed a wide number of autophagic vesicles labelled by monodansylcadaverine after being cultured for 24 hours at 36°C. We hypothesize that stimulation of autophagy may be a requirement for fungal adaptation to the host and for the dimorphic switch from hyphal to yeast growth. To support this assumption, mycelia of Pb18 treated with either rapamycin or 3-MA promoted respectively an increase in the number of MDC-labelled autophagic vesicles or the non-emergence of such vesicles. Moreover, it was shown that the temporal pattern of the emergence of typical structures during M-Y transition, such as the enlargement of the hyphal tips and buds formation, was stimulated by rapamycin and prevented by 3-MA, indicating that dimorphic differentiation was possibly stimulated when autophagy is taking place. Collectively, these data suggest that autophagy may well play a relevant role in the remodelling of the fungus during the thermal dimorphism.

There are no reports on the role of autophagy under any condition in thermally dimorphic fungi. Nevertheless, autophagy appears to play a significant role in filamentous fungi. Not only is it a response mechanism to starvation or other stresses, autophagy is also shown to have an impact on the regulation of fungal growth, morphology, and development, predominantly on the differentiation of cell types [49].

According to Richie et al [50], autophagy in *Aspergillus fumigatus* is required for conidiation and hyphal foraging as a response to nutrient deficiency and is important to the survival of the organism in its native environment. By studying several autophagy-related (*ATG****)*** mutants, Lv et al [51] showed that autophagy plays a critical role in growth, asexual or sexual sporulation and virulence in *Fusarium graminearum*. In the rice blast fungus *Magnaporthe grisea* (*oryzae*) some *ATG* mutants result in an inability to form the infection structure and loss of the ability of the fungus to infect its plant host [26,27,52–54]. A study carried out by Liu et al [55] showed that deletion of the autophagy-related protein MoAtg14 caused a complete loss of its virulence, besides causing problems in conidiation. Moreover, Nadal and Gold [56] demonstrated that the deletion of *ATG1* or *ATG8* in the plant-pathogenic fungus *Ustilago maydis* affected its morphogenesis and resulted in the blockage of the autophagy. Also, a significant reduction of fungal pathogenicity in *Ustilago maydis* was achieved with the *ATG8* mutant [56]. In *Beauveria bassiana*, the deletion of *BbATG1* and *BbATG8* affected the levels of conidial protein BbCP15p, reducing conidiation by approximately 90% and 50%, respectively [57]. Additionally, the virulence in these autophagy-deficient mutant fungi was considerably weakened [57].

Similarly, several cell signalling pathways in yeasts, such as those having the kinase TOR, cAMP, Ras/PKA, Sch9 or Snf1 are related to the regulation of some stage of the autophagy [58–62]. Additionally, all these pathways are associated with cellular glucose levels [63], although each of them is controlled by a distinct set of molecules depending on the nutritional and energetic state of the cell [64].

Kim et al [65] showed that *Aspergillus nidulans* shares some effectors for cellular survival, such as TOR, when subject to rapamycin-induced autophagy and carbon-starvation induced autophagy. Nonetheless, a prolonged cell exposure to carbon deprivation leads to the induction of autophagy by pathways which are not TOR-independent, but PKA-dependent [65].

Considering the results in this work, the autophagy machinery is present also in yeast cells and can be promptly triggered in Pb18 yeasts under different conditions by, for instance, glucose starvation (SD-G), low glucose (SD 0.2%), or rapamycin (SD+R). This latter effect points to the conserved mTORC1 pathway as that involved in the regulation of the fungus autophagy, as it occurs in several eukaryotes [66,67].

Valcourt et al [68] and An et al [69] showed, respectively, that the deprivation of glucose or nitrogen induced morphological changes in yeasts, which became more elongated and presented fewer buds that remained attached to mother cells after cytokinesis. Studies have shown that *Saccharomyces cerevisiae* yeasts undergo increased levels of autophagy, with cell cycle arrest, in response to the starvation, consequently becoming quiescent [68–70]. Conway et al [71] showed that *S*. *cerevisiae* under limited glucose, nitrogen or phosphate, induced a large set of common genes related to growth, cell wall thickening and quiescence.

Regardless of rapamycin inhibition of the nutrient sensor kinase TOR, it should be noted that autophagy in yeasts of *P*. *brasiliensis* was noticed at basal level in a Synthetic medium containing 2% glucose, which should, in theory, favour the activation of either parallel or downstream targets or pathways that could bypass the growth constraints induced by the inhibition of TOR.

It is well known that the activation of the pre-initiation complex depends on both its release from the negative regulation promoted by TORC1 and its activation by AMPK [72]. Nevertheless, under the method applied in this study, it could be expected that AMPK, or AMP-dependent kinase, was inactive in SD medium at initial stages of cell culture when glucose levels are still high and, consequently, AMP/ATP ratio should be favourable to cell growth and proliferation. If such was the case, the incomplete activation of the pre-initiation complex–that is to say, the absence of its inhibition and the lack of its stimulation–should not be sufficient to promote autophagy in *P*. *brasiliensis* in a nutrient-rich environment.

It is not difficult to realize that this model has been little explored and that this is likely due to experimental limitations for a convenient transformation of yeasts, indicating, therefore, we are still far from understanding the details controlling autophagy mechanisms in this fungus. Although the autophagic process is ubiquitous among eukaryotes, the molecular elements that control them in different organisms have already been quite variable, even within Fungi kingdom. For example, in *Cryptococcus neoformans*, 21 genes with homology to the autophagy-related (ATG) genes of *Saccharomyces cerevisiae* [33], which has 34 ATG genes, are known to date [73–76].

This significant difference in the number of players acting on controlling or performing autophagy in these distantly related fungi indicates that in order to elucidate even the general players of autophagy, we need to know a variety of response types that represent the general and specific mechanisms of autophagy control in fungi. Although with limitations, *P*. *brasiliensis* offers a beautiful model of transition between two morphologies: one induced by a simple temperature change and another accompanied by important biochemical modifications that can be easily followed using cell biology and biochemical techniques. This study was the first to show that autophagy occurring in *P*. *brasiliensis* may have a role in the successful establishment of the fungus within its host because it correlates with M-Y dimorphism and is prevented by autophagy inhibitors, thus providing insightful information for future studies aiming at halting paracoccidioidomycosis and other mycoses. Yet, further genetic and *in vivo* analysis might be carried out to ensure that autophagy, in fact, does play a role in host pathogenesis.
